# Open TG-GATEs: a large-scale toxicogenomics database

**DOI:** 10.1093/nar/gku955

**Published:** 2014-10-13

**Authors:** Yoshinobu Igarashi, Noriyuki Nakatsu, Tomoya Yamashita, Atsushi Ono, Yasuo Ohno, Tetsuro Urushidani, Hiroshi Yamada

**Affiliations:** 1 Toxicogenomics Informatics Project, National Institute of Biomedical Innovation, Osaka 567-0085, Japan; 2 Hitachi, Ltd. Information & Telecommunication Systems Company, Government & Public Corporation Information Systems Division, Tokyo 136-8832, Japan; 3 National Institute of Health and Sciences, Tokyo 158-0098, Japan; 4 Department of Pathophysiology, Faculty of Pharmaceutical Sciences, Doshisha Women's College of Liberal Arts, Kyoto 610-0332, Japan

## Abstract

Toxicogenomics focuses on assessing the safety of compounds using gene expression profiles. Gene expression signatures from large toxicogenomics databases are expected to perform better than small databases in identifying biomarkers for the prediction and evaluation of drug safety based on a compound's toxicological mechanisms in animal target organs. Over the past 10 years, the Japanese Toxicogenomics Project consortium (TGP) has been developing a large-scale toxicogenomics database consisting of data from 170 compounds (mostly drugs) with the aim of improving and enhancing drug safety assessment. Most of the data generated by the project (e.g. gene expression, pathology, lot number) are freely available to the public via Open TG-GATEs (Toxicogenomics Project-Genomics Assisted Toxicity Evaluation System). Here, we provide a comprehensive overview of the database, including both gene expression data and metadata, with a description of experimental conditions and procedures used to generate the database. Open TG-GATEs is available from https://toxico.nibiohn.go.jp/english/index.html.

## INTRODUCTION

**Figure 1.**
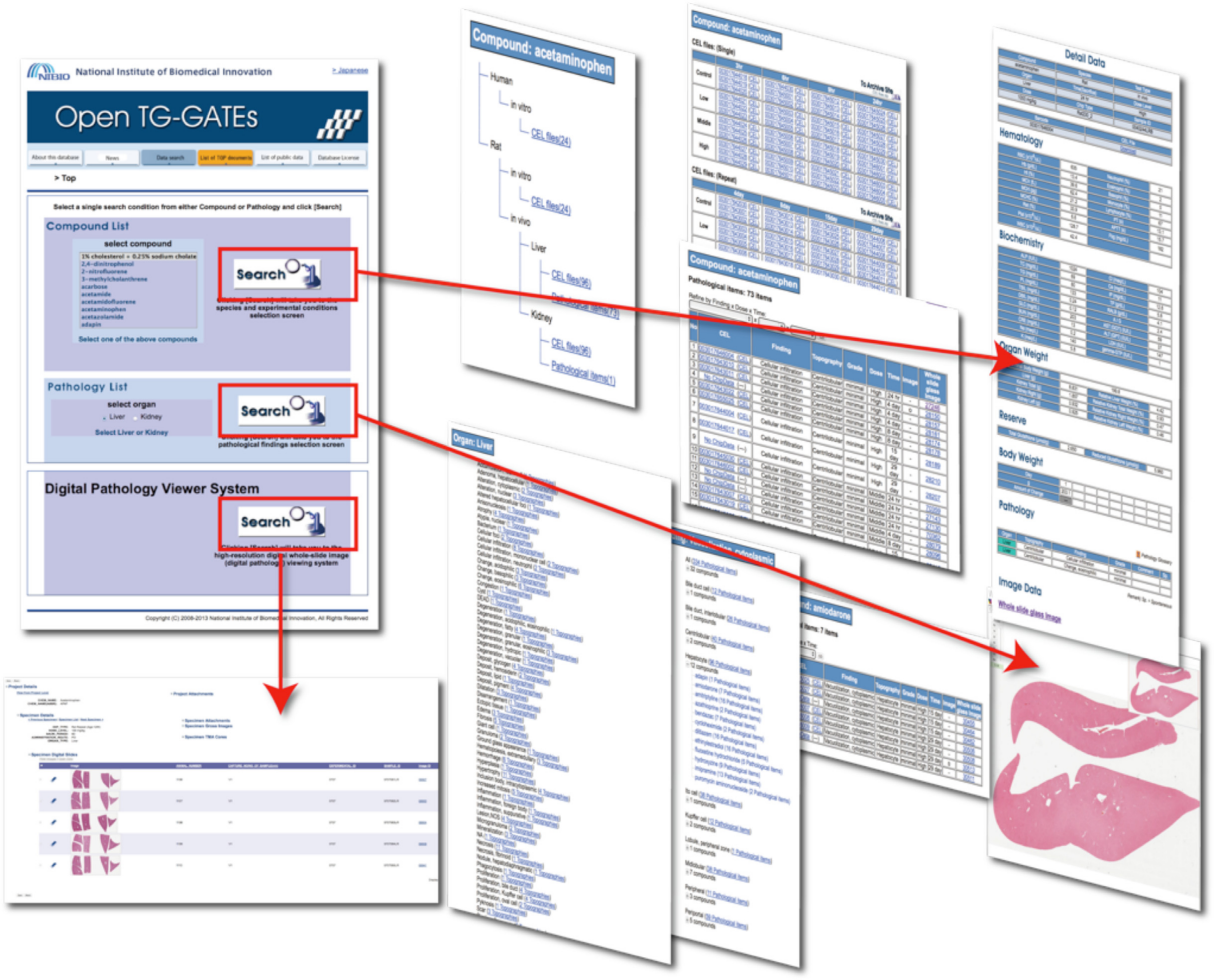
Open TG-GATEs offers hierarchical access from compound and pathology lists to hematology, biochemical parameters and digitized pathology images. Gene expression data are stored as CEL files, which require software capable of interacting with the Affymetrix data file format. Thus, users will have to convert the primary data into a general-purpose format using various algorithms such as MAS5.0, RMA, etc.

**Figure 2.**
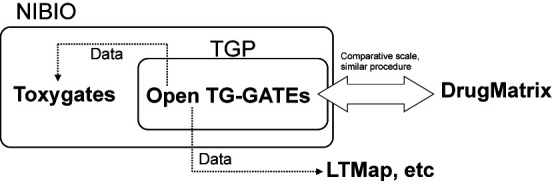
The relationship of the databases and organizations are shown. The dotted line shows the data distribution. NIBIO: National Institute of Biomedical Innovation, TGP: Toxicogenomics project in Japan.

Open Toxicogenomics Project-Genomics Assisted Toxicity Evaluation Systems (TG-GATEs) (Figure 1) is a toxicogenomics database that stores gene expression profiles and traditional toxicological data derived from *in vivo* (rat) and *in vitro* (primary rat hepatocytes, primary human hepatocytes) exposure to 170 compounds at multiple dosages and time points. The toxicology data is composed of biochemistry, hematology and histopathology findings with pathology imaging from the *in vivo* studies and cytotoxicity from the *in vitro* studies. The 170 compounds include representative known liver- and kidney-injuring pharmaceuticals, compounds and chemicals. These data have been generated and analyzed over the course of the 10-year Japanese Toxicogenomics Project (TGP), which was a joint government–private sector project organized by the National Institute of Biomedical Innovation (NIBIO), the National Institute of Health Sciences (NIHS) and 18 pharmaceutical companies (Figure 2).

As specified by relevant regulations, toxicity assessments in the pre-clinical stage of drug development must be conducted using whole animals and cells. In animals, general toxicity in liver and kidney is evaluated with physiological, hematological and biochemical measurements and pathology assessment. In cells, the evaluation of cytotoxicity is conducted by measuring cell viability parameters and morphological changes, often with the use of microscopy. These approaches ensure detection of a certain level of toxicity that might be associated with a given test compound. However, gene expression data is expected to permit the detection of potential toxicities that may not be observable by conventional assessments, thereby facilitating more accurate and predictive decision-making based on toxicity mechanisms (1).

Over the past 10 years, TGP data had been generated at NIHS, NIBIO and several designated contract research organizations using defined standard operating procedures (SOPs). The resulting data were stored, managed and analyzed in a closed version of the database, TG-GATEs. Open TG-GATEs was developed as a publicly available version of the same database, in which the results of 20 118 GeneChip assays are stored along with associated toxicological data and 25 TB of digitized pathology images. Open TG-GATEs is one of the largest public toxicogenomics databases in the world. Using the TG-GATEs data, 36 biomarker sets for specific toxicity mechanisms have to date been defined during project development, and several of these biomarker signatures have been published (2–6).

In the present paper, we describe the data structure and background from a toxicological point of view, along with characteristics of the data relating to experimental conditions. We believe that the data and information in Open TG-GATEs will allow users to gain a greater understanding of toxicity mechanisms and to develop biomarkers for safety assessments of pharmaceutical drug candidates.

## MATERIALS AND METHODS

### Data framework

The gene expression data for a test compound were derived from administration of individual compounds at up to four dose levels and eight time points (corresponding to four single-dose studies and four repeated-dose studies). Studies involving microarray hybridization analysis were performed using three biological replicates of liver and kidney in rat. Biochemistry and hematology data from individual animals were also obtained and stored. The pathology images were digitized and annotated prior to addition to the database. For human and rat primary hepatocytes, test compounds were tested at up to four dose levels and three time points using duplicate microarray hybridization analysis. For primary hepatocytes, cell viability data was also obtained.

### Species and target organ selection

For many years, the rat has been the preferred animal system for pre-clinical toxicological assessment. As a result, more data concerning toxicological mechanisms and endpoints have been accumulated for rat than for any other animal. Therefore, the rat was selected because identification of gene expression changes would have the potential to explain the mechanistic basis of the knowledge accumulated in this animal. Liver is the major organ for metabolism, detoxification of pharmaceuticals and other compounds. Furthermore, clinically significant adverse effects often occur in liver as well as in kidney. *In vitro* experiments in human and rat primary hepatocytes were included for two reasons: first, to provide a bridge between the *in vivo* and *in vitro* data; and second, to permit extrapolation from rat data to human results.

### Compound selection

The tested compounds, shown in Supplementary Table S1, were selected based on literature searches and consensus among pharmaceutical and government toxicologists participating in the TGP. The majority of the compounds were pharmaceuticals with reported liver or kidney toxicity. However, some of the compounds were not pharmaceuticals or known hepato- or nephro-toxins, and instead corresponded to chemicals that had well studied mechanisms of toxicity; these compounds were included as reference chemicals.

### Dose setting

For the *in vivo* studies, the highest dose was selected to match the level demonstrated to induce the minimum toxic effect over the course of a 4-week toxicity study. In principle, the ratio of the concentrations for the low, middle and high dose levels was set as 1:3:10. For the *in vitro* studies, the highest concentration was defined as the dose level yielding an 80–90% relative survival ratio. However, for compounds that dissolved poorly in the vehicle, the highest concentration was defined by the maximum solubility of the compound. In principle, the ratio of the concentrations for the low, middle and high dose levels was 1:5:25.

### Animal treatment

**Figure 3.**
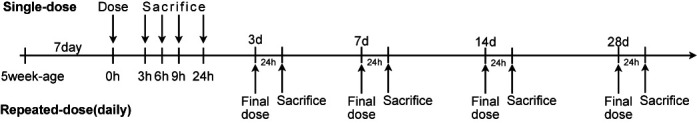
Time lines summarizing the procedures used for *in vivo* studies of single- and repeated-dose toxicity. For the repeated-dose studies, only the final dose is shown.

The experimental procedures for the animal studies have been described previously (7,8) and are summarized here in brief. Animal experiments were conducted by four different contract research organizations. The studies used male Crl:CD Sprague-Dawley (SD) rats purchased from Charles River Japan, Inc. (Hino or Atsugi, Japan) as 5-week-old animals. After a 7-day quarantine and acclimatization period, the animals were allocated into groups of 20 animals each using a computerized stratified random grouping method based on body weight. Each animal was allowed free access to water and pelleted food (radiation-sterilized CRF-1; Oriental Yeast Co., Tokyo, Japan). For single-dose experiments, groups of 20 animals were administered a compound and then fivw animals/time point were sacrificed at 3, 6, 9 or 24 h after administration. For repeated-dose experiments, groups of 20 animals received a single dose per day of a compound and five animals/time point were sacrificed at 4, 8, 15 or 29 days (i.e. 24 h after the respective final administration at 3, 7, 14 or 28 days) (Figure 3). Animals were not fasted before being sacrificed. To avoid effects of diurnal cycling, the animals were sacrificed and necropsies were performed between 9:00 a.m. and 11:00 a.m. for the repeated-dose studies. Blood samples for routine biochemical analyses were collected into heparinized tubes under ether anesthesia from the abdominal aorta at the time of sacrifice.

The experimental protocols were reviewed and approved by the Ethics Review Committee for Animal Experimentation of the NIHS and by the respective contract research organizations.

### Sampling sites of liver and kidney for *in vivo* study

For liver, the sampling site was selected to avoid the hepatic portal vein and choosing a hepatic parenchymal area of the limbic lobe where tissue thickness was consistent. Three tissue fragments were obtained per animal. The center portion was sliced and used as a sample for analysis of pathology. If pathological lesions were identified upon visual inspection, the sampling procedure was repeated at a second location distal from the affected area. For kidney, samples were obtained from the left kidney. Fragments of 1-mm thickness, including a portion of the cortex and medullary regions, were sliced horizontally against the long axis of the kidney using four aligned razors. Three fragments were obtained from each rat. If pathological lesion were identified upon visual inspection, the sampling procedure was repeated using the right kidney.

### Hepatocyte treatment

Human cryopreserved hepatocytes were purchased from Tissue Transformation Technologies, Inc. (Edison, NJ, USA) and CellzDirect, Inc. (Pittsboro, NC, USA). Six lots of human hepatocytes were used during the course of the project. The lot information is shown in Supplementary Table S2. Rat primary-cultured hepatocytes were prepared as described previously (9), and were derived from 5-week-old male SD rats that had been subjected to a 5-day observation period after arrival. The lot information of the rat primary-cultured hepatocytes is provided in Supplementary Table S3. The detailed procedure for the measurement of cell viability parameters is described in the Supplementary information.

### GeneChip analysis

The experimental procedure for the analysis of gene expression has been described previously (7,8), with the pertinent points briefly summarized here. In general, we selected rats of moderate body weight (the middle three of five animals). All CEL data in Open TG-GATEs passed quality control (QC). QC was performed at each step of the sample preparation and at the image-scanning step of GeneChip analysis. A collection of analytical information was checked as the final QC step, including background signal, corner signal, number of presence/absence calls and the expression values of housekeeping genes. The intra- and inter-group reproducibility was also evaluated. If a sample was found to be damaged, it was replaced by one of the two remaining fragments. The QC information is not open to the public. During the course of the 10-year project, two kinds of kits were used in the biotin-labeled cRNA synthesis procedure. These kits were the BioArray High Yield RNA Transcription Labeling Kit (Enzo Diagnostics, Farmingdale, NY, USA) and the GeneChip^®^ IVT Labeling kit (Affymetrix, Santa Clara, CA, USA). For rat and human samples, the Rat Genome 230 2.0 Array and the Human Genome U133 Plus 2.0 Array were used, respectively. The probe set information is obtained from the Affymetrix support materials page, including gene names and identifiers in other representative databases. The probe set information is typically updated once a year. Gene expression analysis, including data QC processes, was performed according to an SOP, which for the most part followed the procedures supplied by the respective kit manufacturers.

### Metadata in Open TG-GATEs

The metadata in the database includes the code for each GeneChip and corresponding data such as test compound name, dosage and body weight, as well as hematological and biochemical data (Table 1). The metadata files are stored in ‘Attribute.tsv’ for each compound. Peer-reviewed histopathological findings and topography information are also provided in Open TG-GATEs and the archived site (http://dbarchive.biosciencedbc.jp/en/open-tggates/download.html). Food consumption data is available only from the archived site.

**Table 1.**
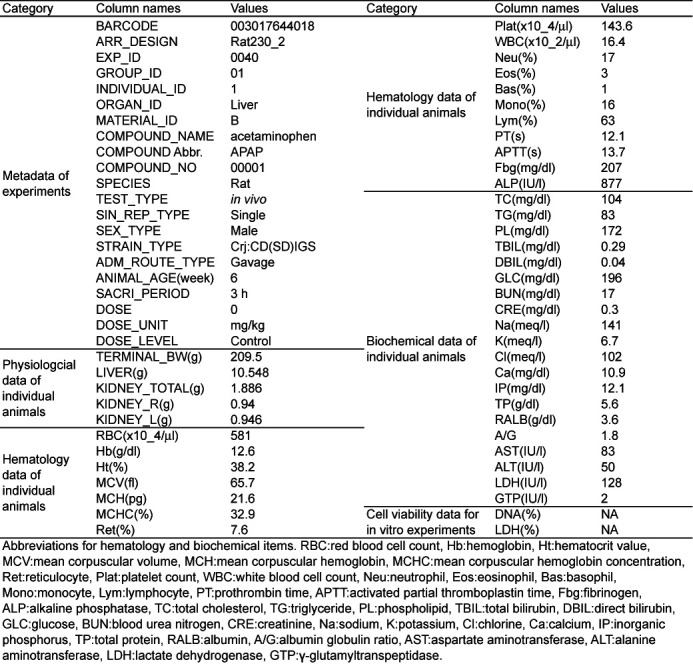
The column names and example data in attribute file are shown

### Pathology images and annotation

The liver and kidney sections were stained with H&E (hematoxylin and eosin) and mounted on glass slides. Images of the sections were converted to digital pathology images using ScanScope AT (Aperio Technologies Inc., CA, USA). The digital images were saved and stored in .svs format, which consists of TIFF format files with associated sample dimensions and other relevant values. The pathological information is composed of histopathological finding, topography and grade. These curated data were originally annotated by each contract research organization and the data were subsequently peer-reviewed by the pathologists of the TGP member companies. The annotation was conducted based on a ‘Pathology Glossary’, a consensus controlled vocabulary for histopathological findings for liver and kidney (https://toxico.nibiohn.go.jp/open-tggates/doc/pathology_parameter.pdf), which was originally assembled by NIHS. Representative cropped pathology images were assembled in a pdf (‘3. Histopathology photograph collection/list of histopathology findings’ at https://toxico.nibiohn.go.jp/english/seika.html). Unfortunately, the XY positions of the cropped images from the full-size digital pathology image were not recorded.

## DISCUSSION

The term toxicogenomics appeared in publications starting in the late 1990s (10). The concept was expected to enhance the determination of chemical toxicity by improving the prediction and understanding of the mechanisms of toxicity. Before the initiation of TGP, Iconix Pharmaceuticals, Inc. (11), had developed its own toxicogenomics database. This dataset is currently available to the public as the DrugMatrix database of the National Toxicology Programs of the National Institute of Environmental Health Sciences (https://ntp.niehs.nih.gov/drugmatrix/index.html). Other toxicogenomic and related databases are also available, including Chemical Effects in Biological Systems (12) and the Comparative Toxicogenomics database (13).

The Open TG-GATEs was developed in an effort to be a ‘gate’ to a new frontier beyond toxicogenomics and other fields. Therefore, the gene expression data is stored in the CEL format in order to allow users to select suitable methods (14–16) to covert CEL files to numerical data. At the same time, this file type may render the data difficult to analyze for users unfamiliar with Affymetrix data analysis. To address this challenge, Nystrom *et al.* have made the data available as a second-party web service called Toxygates (17). The Toxygates site is administered independently from Open TG-GATEs and offers data analysis functionality using preprocessed data obtained from Open TG-GATEs (Figure 2).

To date, one of the major barriers in computational toxicology has been the limited number of public toxicity datasets upon which computational models could be built (18). After the Open TG-GATEs and DrugMatrix data were made available to public, the challenge has become focused on data mining. In addition to data mining, validation will be required at some stage. There are two ways to validate analyses, results or models using Open TG-GATEs data. One way is to (re)-produce the gene expression data using the same experimental conditions and procedures described in this paper. Another way is to use the DrugMatrix database where similarities exist in the compounds and models tested. We believe the DrugMatrix database is the most suitable database for comparison with the Open TG-GATEs data, particularly in consideration of its scale, the wide variety of compounds, animal strains and type of GeneChip (Figure 2).

Since the data was made publicly available, a significant number of studies and reviews have been conducted by agencies/groups outside of the TGP. The first comprehensive review of a comparison of DrugMatrix and Open TG-GATEs was written by Chen *et al.* (19). Zhang *et al.* successfully identified a consensus gene network between *in vivo* and *in vitro* studies (20). The Open TG-GATEs and DrugMatrix data was used as reference data in a toxicogenomics analysis using RNA-seq with text-mining (21). A carcinogenicity prediction study was conduced using human *in vitro* data with a connectivity map (22). Recently, a web-based tool that assesses potential liver toxicity using specific gene signatures was also developed (23). It also should be noted that Open TG-GATEs data was utilized as trial data in CAMDA (Critical Assessment of Massive Data Analysis) 2012 (http://camda.bioinfo.cipf.es/camda2012/) and 2013 (http://dokuwiki.bioinf.jku.at/doku.php).

A new and prominent feature in this database is the massive amount of digital pathology images. We think the digital pathology images also have the potential to improve toxicity assessments by their use as phenotypic end points. One of the great challenges in this field is to create an automatic pathology annotation system for liver and kidney toxicity. This will greatly improve the efficiency of current pathology diagnosis. Even before this is accomplished, the extraction of further parameters, such as the degree, extent and location of injury, and their associated gene expression profiles and toxicological data, would enable more accurate toxicity predictions.

There are some important points to consider when analyzing Open TG-GATEs data, several of which are discussed here. The first point is concerned with the gene expression data obtained from *in vitro* experiments. When a test compound with direct cytotoxicity in hepatocytes is applied to cells, mRNA expression is drastically changed (1). In such cases, even after global normalization, the fold-change values (differential expression between treated and control samples) will not reflect actual fold-changes. Secondly, it is important to note that the concentration of some compounds (*in vitro* experiments) will not achieve the toxic doses because of limited solubility in the growth medium. In such cases, gene expression will not exhibit significant alterations. A third concern is that of potential variations in methodology. TGP hematology and biochemical parameters were generated over the course of 10 years among different contract research organizations. Thus, the instruments and reagents used to obtain the data are not likely to remain identical over a 10 year period, even within a single organization. Therefore, analyses comparing parameters across the entire database may exhibit certain systematic trends. Consequently, the use of individual experimental sets is recommended for conducting rigorous analyses.

As mentioned above, the Open TG-GATEs data has already been utilized by third-party groups. At the same time, we have started to receive questions about detailed aspects of the data. Although some of the answers to these inquiries have already been published, they may be dispersed in publications published over the previous 10 years, while other answers remain unpublished. In order to understand the characteristics and structure of the data and avoid misuse, we have attempted to provide here an overview of Open TG-GATEs as well as some general guidelines for use of the database.

## FUTURE DIRECTIONS

Although toxicogenomics using transcriptomics data is a powerful approach to identify gene set signatures for toxicity, we recognize that a multi-omics approach, such as in combination with metabolomics data, can facilitate the interpretation of gene expression changes due to its complementary role to transcriptomics data (24). We plan to integrate metabolomics data and leverage its complementary relationship in the future. Currently, we are focusing on the efficacy and safety of adjuvants that are components of various vaccines. Similar protocols and methodologies are being applied to obtain gene expression profiles and toxicology data related to adjuvants. These data will be open to the public in a format that can be integrated with Open TG-GATEs data to assess the efficacy and safety of adjuvants.

## SUPPLEMENTARY DATA

Supplementary Data are available at NAR Online.

## References

[1] Urushidani T., Sahu SC (2007). Prediction of hepatotoxicity based on the toxicogenomics database. Hepatotoxicity: From Genomics to In Vitro and In Vivo Models.

[2] Kondo C., Minowa Y., Uehara T., Okuno Y., Nakatsu N., Ono A., Maruyama T., Kato I., Yamate J., Yamada H. (2009). Identification of genomic biomarkers for concurrent diagnosis of drug-induced renal tubular injury using a large-scale toxicogenomics database. Toxicology.

[3] Gao W., Mizukawa Y., Nakatsu N., Minowa Y., Yamada H., Ohno Y., Urushidani T. (2010). Mechanism-based biomarker gene sets for glutathione depletion-related hepatotoxicity in rats. Toxicol. Appl. Pharmacol..

[4] Uehara T., Minowa Y., Morikawa Y., Kondo C., Maruyama T., Kato I., Nakatsu N., Igarashi Y., Ono A., Hayashi H. (2011). Prediction model of potential hepatocarcinogenicity of rat hepatocarcinogens using a large-scale toxicogenomics database. Toxicol. Appl. Pharmacol..

[5] Minowa Y., Kondo C., Uehara T., Morikawa Y., Okuno Y., Nakatsu N., Ono A., Maruyama T., Kato I., Yamate J. (2012). Toxicogenomic multigene biomarker for predicting the future onset of proximal tubular injury in rats. Toxicology.

[6] Yamada F., Sumida K., Uehara T., Morikawa Y., Yamada H., Urushidani T., Ohno Y. (2012). Toxicogenomics discrimination of potential hepatocarcinogenicity of non-genotoxic compounds in rat liver. J. Appl. Toxicol..

[7] Hirode M., Ono A., Miyagishima T., Nagao T., Ohno Y., Urushidani T. (2008). Gene expression profiling in rat liver treated with compounds inducing phospholipidosis. Toxicol. Appl. Pharmacol..

[8] Uehara T., Ono A., Maruyama T., Kato I., Yamada H., Ohno Y., Urushidani T. (2010). The Japanese toxicogenomics project: application of toxicogenomics. Mol. Nutr. Food Res..

[9] Sumida K., Igarashi Y., Toritsuka N., Matsushita T., Abe-Tomizawa K., Aoki M., Urushidani T., Yamada H., Ohno Y. (2011). Effects of DMSO on gene expression in human and rat hepatocytes. Hum. Exp. Toxicol..

[10] Nuwaysir E.F., Bittner M., Trent J., Barrett J.C., Afshari C.A. (1999). Microarrays and toxicology: the advent of toxicogenomics. Mol. Carcinog..

[11] Engelberg A. (2004). Iconix Pharmaceuticals, Inc.–removing barriers to efficient drug discovery through chemogenomics. Pharmacogenomics.

[12] Waters M., Stasiewicz S., Merrick B.A., Tomer K., Bushel P., Paules R., Stegman N., Nehls G., Yost K.J., Johnson C.H. (2008). CEBS-Chemical effects in biological systems: a public data repository integrating study design and toxicity data with microarray and proteomics data. Nucleic Acids Res..

[13] Davis A.P., Murphy C.G., Johnson R., Lay J.M., Lennon-Hopkins K., Saraceni-Richards C., Sciaky D., King B.L., Rosenstein M.C., Wiegers T.C. (2013). The comparative toxicogenomics database: update 2013. Nucleic Acids Res..

[14] Hubbell E., Liu W.-M., Mei R. (2002). Robust estimators for expression analysis. Bioinformatics.

[15] Irizarry R.A., Bolstad B.M., Collin F., Cope L.M., Hobbs B., Speed T.P. (2003). Summaries of Affymetrix GeneChip probe level data. Nucleic Acids Res..

[16] McCall M.N., Bolstad B.M., Irizarry R.A. (2010). Frozen robust multiarray analysis (fRMA). Biostatistics.

[17] Nyström-Persson J., Igarashi Y., Ito M., Morita M., Nakatsu N., Yamada H., Mizuguchi K. (2013). Toxygates: interactive toxicity analysis on a hybrid microarray and linked data platform. Bioinformatics.

[18] Cronin M.T.D. (2013). Computational toxicology is now inseparable from experimental toxicology. Altern. Lab. Anim..

[19] Chen M., Zhang M., Borlak J., Tong W. (2012). A decade of toxicogenomic research and its contribution to toxicological science. Toxicol. Sci..

[20] Zhang J.D., Berntenis N., Roth A., Ebeling M. (2014). Data mining reveals a network of early-response genes as a consensus signature of drug-induced in vitro and in vivo toxicity. Pharmacogenomics J..

[21] Yu K., Gong B., Lee M., Liu Z., Xu J., Perkins R., Tong W. (2014). Discovering functional modules by topic modeling RNA-Seq Based Toxicogenomic Data. Chem. Res. Toxicol..

[22] Caiment F., Tsamou M., Jennen D., Kleinjans J. (2014). Assessing compound carcinogenicity in vitro using connectivity mapping. Carcinogenesis.

[23] Xing L., Wu L., Liu Y., Ai N., Lu X., Fan X. (2014). LTMap: a web server for assessing the potential liver toxicity by genome-wide transcriptional expression data. J. Appl. Toxicol..

[24] Uehara T., Horinouchi A., Morikawa Y., Tonomura Y., Minami K., Ono A., Yamate J., Yamada H., Ohno Y., Urushidani T. (2014). Identification of metabolomic biomarkers for drug-induced acute kidney injury in rats. J. Appl. Toxicol..

